# Chemogenetic inhibition of prefrontal projection neurons constrain top-down control of attention in young but not aged rats

**DOI:** 10.1007/s00429-021-02336-2

**Published:** 2021-07-10

**Authors:** Michael R. Duggan, Surbhi Joshi, Jacob Strupp, Vinay Parikh

**Affiliations:** Department of Psychology and Neuroscience Program, Temple University, Philadelphia, PA 19122

**Keywords:** prefrontal cortex, aging, attention, DREADDs, cholinergic

## Abstract

The prefrontal cortex (PFC) governs top-down control of attention and is known to be vulnerable in aging. Cortical reorganization with increased PFC recruitment is suggested to account for functional compensation. Here, we hypothesized that reduced PFC output would exert differential effects on attentional capacities in young and aged rats, with the latter exhibiting more robust decline in performance. A chemogenetic approach involving Designer Receptors Exclusively Activated by Designer Drugs was utilized to determine the impact of silencing PFC projection neurons in rats performing an operant attention task. Visual distractors were presented in all behavioral testing sessions to tax attentional resources. Under control conditions, aged rats exhibited impairments in discriminating signals with the shortest duration from non-signal events. Surprisingly, chemogenetic inhibition of PFC output neurons did not worsen performance amongst aged animals. Conversely, significant impairments in attentional capacities were observed in young subjects following such manipulation. Given the involvement of PFC-projecting basal forebrain cholinergic neurons in top-down regulation of attention, amperometric recordings were conducted to measure alterations in prefrontal cholinergic transmission in a separate cohort of young and aged rats. While PFC silencing resulted in a robust attenuation of tonic cholinergic signaling across age groups, the capacity of generate phasic cholinergic transients was impaired only amongst young animals. Collectively, our findings suggest reduced efficiency of PFC-mediated top-down control of attention and cholinergic system in aging, and that activity of PFC output neurons does not reflect compensation in aged rats, at least in the attention domain.

## Introduction

Attentional processes are fundamental to the deployment of cognitive resources for goal-directed learning, memory and executive functions (Sarter et al., 2003; Sarter et al., 2005). The prefrontal cortex (PFC) is integral to the control of cognitive functions, including attention. Substantial evidence from studies in rodents and non-human primates indicate that transection or excitotoxic lesions of PFC subregions impair performance in tasks of attention (Kahn et al., 2012; Passetti et al., 2002; Rossi et al., 2009; Suzuki & Gottlieb, 2013). Specifically, damage to the PFC disrupts the ability to switch top-down control of attention and increases distractibility, which is consistent with lesion studies in humans (Chao & Knight, 1995; Mesulam, 1981). Additionally, electrophysiology and neuroimaging studies demonstrate PFC-driven modulation of neuronal activity in the parietal and visual cortices in tasks of visual attention, further supporting the involvement of PFC in top-down modulation of attention (Gazzaley et al., 2007; Lee & D’Esposito, 2012; Paneri & Gregoriou, 2017).

Neuroanatomical mapping studies illustrate that cholinergic neurons residing in the basal forebrain (BF), encompassing the areas of nucleus basalis, substantia innominate, and horizontal diagonal band of Broca, project to multiple spatially distinct areas in PFC that are functionally connected, some of which maintain reciprocal afferents to their distal counterparts in the BF (Bloem et al., 2014; Zaborszky et al., 2015). Removal of cholinergic inputs from the medial PFC (mPFC) reduces choice accuracies and produces speed-accuracy tradeoffs under conditions of distraction as well as modality uncertainties in various tasks of attention (Dalley, Theobald, et al., 2004; Maddux et al., 2007; Newman & McGaughy, 2008). Studies employing microdialysis and electrochemical approaches in task-performing rats demonstrate a robust association between prefrontal cholinergic activity and cognitive operations required to sustain attentional performance (Gritton et al., 2016; Howe et al., 2013; Parikh & Sarter, 2008). Moreover, the PFC has also been shown to regulate cholinergic transmission in downstream cortical targets (such as the parietal cortex) to optimize input processing and maintain ensuing attentional performance (Broussard et al., 2009; Nelson et al., 2005). Thus, bidirectional interactions between the PFC and BF cholinergic system are thought to induce synergistic neuronal responses to ethologically relevant stimuli, coordinate downstream neuronal circuits, and modulate top-down attention (Hasselmo & Sarter, 2011; Katsuki & Constantinidis, 2013).

The decline in top-down attention is well documented in aging (Madden, 2007; Quigley & Müller, 2014). Moreover, attentional deficits are proposed to contribute to progressive impairments in executive and mnemonic functions in Alzheimer’s disease (AD), a neurodegenerative disorder prominent in older adults (Perry & Hodges, 1999). Therefore, it is important to delineate neural mechanisms underlying age-related changes in top-down regulation of attention.

The structure and functions of the PFC are highly vulnerable to the effects of aging (Gallagher & Rapp, 1997; Samson & Barnes, 2013). However, a plethora of functional neuroimaging investigations in humans demonstrate that elevated PFC activity in older adults positively correlates with cognitive performance, but negatively correlates with age-related decreases in occipital activity (Cabeza et al., 2004; Davis et al., 2008; Grady et al., 1994). Moreover, age-related increases in PFC activity are also found to be proportional to task difficulty itself, with aged individuals requiring more activation as demands on attention increase (Ansado et al., 2012; Reuter-Lorenz & Cappell, 2008; Reuter-Lorenz & Lustig, 2005). Together, these studies suggest the elevated recruitment of PFC networks could reflect an adaptive reorganization process, enabling an individual to compensate for the declining sensory and information processing that would otherwise constrain attentional capacities in aging. Contrary to this view, prefrontal overactivation in aging has not been consistently associated with improved performance (Rypma et al., 2007; Stevens et al., 2008; Zarahn et al., 2007). Thus, it remains unclear whether PFC reorganization is reflective of beneficial or detrimental cognitive changes in aging.

Here, we hypothesized that silencing of PFC projection neurons would exert differential effects on attentional capacities and cholinergic signaling in young and aged rats, with the latter exhibiting a more robust decline in performance and ACh release. Utilizing the Designer Receptors Exclusively Activated by Designer Drugs (DREADD) approach, we selectively targeted excitatory projection neurons of the mPFC in rats using an adeno-associated viral (AAV) vector that expresses inhibitory G protein-coupled muscarinic receptors (hM4Di; Gi-DREADD) fused with the mCherry reporter under the control of Calmodulin Kinase II alpha (CaMKIIa) promoter. The suppression of prefrontal output neuronal activity was induced via administration of the pharmacologically inert ligand clozapine-N-oxide (CNO), which possesses high affinity for such engineered G protein-coupled receptors. To assess performance, an operant attention task that requires subjects to detect a visual signal of varying durations under distracting conditions and that engages top-down attentional control mechanisms was used. Electrochemical recordings were conducted to assess the impact of chemogenetic silencing on cholinergic transmission in the PFC. Contrary to our postulation, young but not aged animals were more susceptible to performance decrements under distracting conditions and cholinergic disruption following such a manipulation. Our findings suggest reduced efficiency of PFC-mediated top-down control of attention and cholinergic system in aging, and that activity of PFC output neurons does not reflect compensation in aged rats, at least in the attention domain.

## Methods

### Animals

Male Wistar rats 2–3 months old (young) or 10–12 months (retired breeders) were acquired from Charles River laboratories (Malvern, PA, USA). Animals were double-housed in standard conditions with humidity (40–50%), temperature (18–21°C) and light cycle (12-h light/dark cycle, lights on at 7:00AM) controlled. Retired breeders were maintained until 22 months of age and grouped as “aged” rats prior to the initiation of experiments. For operant behavior studies (see description below), rats were partially water-deprived by restricting access to a 10-min period in the home cage following each behavioral session. Training took place 6 days/week and on non-training days, water access was increased to 20 min. Food was available to animals *ad-libitum* during behavioral studies. All young and aged rats were single-housed a week prior to the initiation of water regulation and operant behavior. Single housing was maintained throughout the duration of behavioral training and testing. A separate cohort of naïve young and aged rats was used for experiments involving *in vivo* amperometric recordings. All experiments were conducted in accordance with National Institute of Health guidelines and were approved by the Institutional Animal Care and Use Committee, as well as the Institutional Biosafety Committee at Temple University. A total of 50 rats were used in our study. 3 aged rats were removed due to either deteriorating health or mortality following surgeries.

### Operant Behavior Procedures

#### Apparatus

Animals were trained in modular operant chambers enclosed in sound-attenuating boxes, with each box containing a fan to provide ventilation as well as discrete background noise (Med Associates, Inc., St. Albans, VT, USA). Each chamber had two retractable levers, a central panel with three panel lights (2.8 W), a liquid spigot attached to a water dispenser, and a house light (2.8W) located on the rear wall. All events (e.g. signal delivery, lever presentation, water dispense) were written in Medstate notation programs and communicated via the SmrtCtrl interface running the Med-PC software on a Dell Optiplex 960 computer.

#### Sustained Attention Task (SAT)

**Figure 1.**
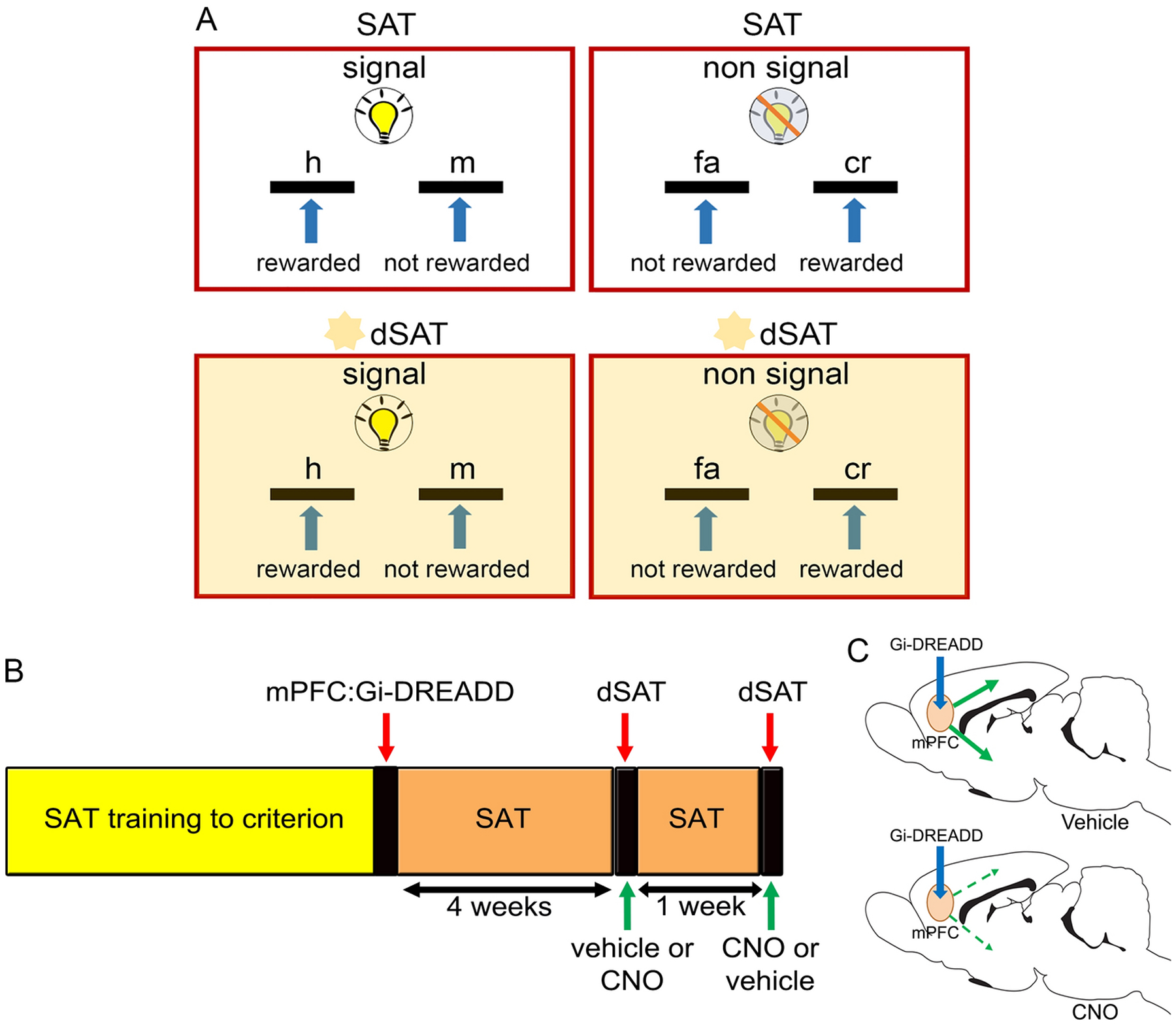
Schematic of the operant task procedures and experimental design. A) An illustrated representation of the sustained attention task (SAT). Rats, pseudo-randomly assigned to a lever (either left or right), discriminated between signal (illumination of the central panel light) and non-signal (no illumination) trials by pressing a lever (either left or right). For example, for a subject assigned to the left lever, a left lever press on a signal trial was scored as a hit (h) and rewarded, while a right lever press on a signal trial was scored as a miss (m) and not rewarded. Conversely, for the same animal on non-signal trials, a right lever press was scored as a correct rejection (cr) and rewarded, while a left lever press was considered a false alarm (fa) and not rewarded. The assignment of lever (left or right) for each trial type was counterbalanced across animals within a group. For final testing, attentional performance was assessed under challenging conditions in the distractor version of the task (dSAT). Here, animals were presented with visual distractors (flashing house light @ 0.5 Hz) during the second block of the behavioral session. B) Schematic of the experimental design. Young and aged rats trained to criterion, received bilateral infusions of AAV vector expressing hM4D(Gi) and the reporter gene mCherry under the control CaMKIIa promoter into the medial prefrontal cortex (mPFC). Following surgery, rats were placed back on the SAT for 4 weeks, at which point the animals were given a systemic injection of either vehicle or CNO and completed a dSAT testing session. A second dSAT testing session was given a week later to the same animals with the sequence of injections reversed. Given the within-subjects nature of the experimental design, counterbalancing was employed with regards to the order of injection type. C) Sagittal brain view depicting the intact (vehicle) or suppressed (CNO) prefrontal output following chemogenetic manipulation.

Water-regulated young and aged rats were trained in an operant-based sustained attention task (SAT) as previously described (Demeter et al., 2008; McGaughy & Sarter, 1995; St Peters et al., 2011; Yegla & Parikh, 2017). Animals were initially autoshaped using an FR-1 reinforcement schedule to attain the lever press response and succeeding water reward (0.02 mL). To deter bias towards one lever over another, lever presses on the dominant lever (i.e. the lever with > 5 presses) were not reinforced until such a bias was reduced. After attaining 120 lever presses on a given behavioral session, animals were moved onto the next phase of training where animals were required to discriminate between signal (illumination of the central panel light for 1s) and non-signal (no illumination of the central panel) events. Two seconds after a given event, both levers were extended and remained available for 4s or until a lever press occurred. If no response occurred, an omission was recorded and the intertrial interval (12 ± 3s) was reinstated. Rats were pseudo-randomly designated to a lever side under signal trial conditions and had to respond on the opposite lever for non-signal trials. For example, if a rat was designated to the left lever, on a signal trial, a left lever press was rewarded (i.e., hit; h) but not a right lever press (i.e., miss; m). In contrast, a right lever press on a non-signal trial was rewarded (i.e., correct rejection; cr) but left lever press (i.e., false alarm; fa) was not rewarded (see SAT illustration in Fig 1A). The presentation of signal and non-signal events was pseudo-randomized and counterbalancing was employed such that half of the animals were trained with the reverse set of rules.

After attaining performance criterion (i.e. ≥70% correct responses on both signal and non-signal trials over three consecutive days), animals were advanced to the last stage of behavioral training. Here, several modifications to the paradigm further constrained an animal’s capacity to continuously monitor the central panel; specifically, the duration of the signal was decreased to 25ms, 50ms, or 500ms, the intertrial interval was reduced to 9 ± 3s, and the house light remained illuminated throughout a given session. At this stage of training, each behavioral session consisted of 81 signal (27 trials per signal duration) and 81 non-signal trials. Each behavioral session was divided into three blocks of 54 trials (27 signal and 27 non-signal trials) such that each duration of signal was presented 9 times per block. After reaching criterion (i.e. ≥70% correct responses on 500ms trials, ≥70% correct responses on non-signal trials, and ≤20% omissions over three consecutive days) animals were prepared for stereotaxic surgeries for viral vector infusions (see procedure below). For final testing, animals were exposed to the distracter version (dSAT) of the task that involved the presentation of visual distractors (flashing house light @ 0.5 Hz) during the second block of the behavioral session (Fig 1A). The discrimination of weaker target signals from the non-signal events in the presence of salient visual distractors in the dSAT engages top-down attentional control mechanism (Demeter et al., 2008; Nuechterlein et al., 2009). All animals were exposed to the distractors once prior to stereotaxic surgeries for AAV vector infusions (see section on “Stereotaxic surgeries for vector infusions and experimental design). This procedure was adopted to minimize the novelty effects of distractors while evaluating the effects of chemogenetic silencing of prefrontal projection neurons in young and aged rats on performance in the dSAT sessions.

#### Behavioral Measures

The total numbers of hits, misses, correct rejections, false alarms, and omissions were recorded for the entire behavioral session. Each session was analyzed in terms of the proportion of hits (h = h/[h+m]*100) for each signal length, proportion of correct rejections (cr = cr/[cr+fa]*100), and proportion of omissions ([omitted trials/total trials]*100. The overall measure of attentional performance was calculated as a performance score (SAT/dSAT score) using the formula: (h − fa)/[2(h + fa) − (h + fa)^2^] as described previously (Demeter et al., 2008; McGaughy & Sarter, 1995; St Peters et al., 2011; Yegla & Parikh, 2017). SAT/dSAT scores vary from +1.0 to −1.0; a score of +1.0 indicated that all responses on signal and non-signal trials were correct, 0 indicated the inability to discern signal from non-signal events, and −1.0 indicated that all responses were misses/false alarms. Performance scores were calculated for each signal duration and for the entire session. Performance measures were also calculated for each task block of 54 trials.

### Stereotaxic surgeries for vector infusions and experimental design

All surgeries were conducted under aseptic conditions. Anesthesia was induced with isoflurane (4–5%) using an anesthesia machine (Surgivet, Dublin, OH). Young and aged rats trained to SAT criterion were placed in a stereotaxic frame (Model # 962; David Kopf Instruments, Tujunga, CA) and the head was positioned into the head frame using ear bars. Anesthesia was maintained throughout the surgical procedure with 2–3% isoflurane using oxygen as a carrier gas at a flow rate of 1 mL/min. An isothermal deltaphase pad (Braintree Scientific, Braintree, MA, USA) was used to consistently maintain animals’ body temperature at 37°C throughout the surgical procedure. For DREADD studies, young (N = 8) and aged (N = 9) rats trained to SAT criterion received bilateral infusions of AAV8-CaMKIIa-hM4D(Gi)-mCherry (Addgene viral prep # 50477-AAV8; vector plasmid was a gift from Dr. Bryan Roth) into the mPFC (AP: +3.0mm, ML: −0.7mm, DV: −2.7mm from dura). We attempted to target the anterior cingulate, prelimbic, and infralimbic areas with the DREADD vector. These regions constitute the dorsomedial and ventromedial components of the mPFC in rodents and are known to be critically involved in mediating different aspects of attention. For instance, anterior cingulate corresponds to the dorsomedial axis and is involved in reducing distraction and allocating attentional resources towards goals over prolonged periods of time (Dalley, Cardinal, et al., 2004; Kim et al., 2016; Newman & McGaughy, 2011). On the other hand, the ventromedial subregions (i.e. prelimbic and infralimbic areas) are implicated in executive or decisional aspects of attention (Chudasama & Muir, 2001; Passetti et al., 2002). Together, these mPFC subregions are suggested to orchestrate top-down regulation of sustained attention (Luchicchi et al., 2016). AAV vector infusions (0.4 μL/hemisphere) were made using a 10-μl Hamilton syringe at a rate of 0.1μl/min and the needle remained in place for an additional 4min to allow for complete diffusion of virus particles. Following surgery, rats were allowed to recover for a period of 72h and then placed back on the SAT for 4 weeks; at this point, animals were given a systemic injection of either vehicle (5% DMSO in 0.9% saline) or Clozapine-N-oxide dissolved in vehicle (CNO; 1mg/kg i.p.) and then subjected to a dSAT testing session 60-min later. A second dSAT testing session was given a week later to the same animals with the sequence of injections reversed (see Fig 1B for the schematic of experimental design). Given the within-subjects nature of the experimental design, counterbalancing was employed with regards to the order of injection type. Animals were maintained on SAT between the two dSAT testing sessions. Following the last behavioral session, animals were perfused for immunohistochemical examination of the virus spread and transduction efficiency (see procedure below). To determine whether the behavioral effects of CNO are Gi-DREADD-specific, another cohort of young rats (N = 7) trained on SAT received bilateral infusions of a control AAV vector expressing only the reporter mCherry under CaMKII promoter pAAV-CaMKIIa-mCherry (Addgene viral prep #114469-AAV5; vector plasmid was a gift from Dr. Karl Diesseroth).

For functional validation studies assessing the efficacy of Gi-DREADD, a separate cohort of performing (N = 6) and non-performing (N = 6) rats were used. These studies were conducted in young animals. Performing rats were trained in SAT and following the attainment of criterion, received bilateral infusions of Gi-DREADD vector into the mPFC as described above. Animals remained on SAT 4-weeks post-surgery following which they were tested on dSAT. Half of the animals received vehicle (N = 3) while the other half received CNO (N = 3) 60-min prior to behavioral testing. Non-performing rats were exposed to the operant chambers and underwent the same Gi-DREADD vector manipulations as their performing counterparts but were never trained on SAT. On the testing day, these animals were administered with either vehicle (N = 3) or CNO (N = 3) and placed in the operant box 60-min later. These animals remained in the box for a time period that was consistent with performing rats (~40 min). All rats were perfused 60-min following completion of the behavioral testing. Brains were isolated for semi-quantitative double immunohistochemistry to determine Gi-DREADD-induced reduction of performance-associated activity of prefrontal neurons as described below.

### Immunohistochemistry and image analysis

Animals were transcardially perfused using 100 mL of ice-cold 0.1 M PBS followed by 300 mL of 4% paraformaldehyde (PFA; pH 7.4–7.6). Brains were removed, postfixed overnight in PFA and then transferred to 30% sucrose (in 0.1 M PBS) for 72 h. Coronal sections (50μm) were taken on a freezing microtome (SM2000R, Leica, Wetzlar, Germany) and the slices were stored in cryoprotectant solution (15% glucose, 30% ethylene glycol, and 0.04% sodium azide in 0.05 M PBS) at −20 °C until further processing.

Brain slices from the mPFC were stained and processed to assess transduction efficiency of AAV vectors by conducting m-Cherry/CamKII double immunostaining with slight modifications from the procedure described earlier (Wang et al., 2018). Serial sections randomly selected from the rostral-caudal axis of PFC (AP: +3.2 – +2.7 mm) were thawed and rinsed in 0.05 M Tris-buffered saline (TBS). Following 1 h blocking in 10% donkey serum, sections were incubated in mouse anti-CAMKII antibody (ThermoFisher, 1:1,000 dilution) overnight at 4°C on a shaker. The sections were washed (3 × 5 min) in TBS containing 1% triton X 100 (TBST) and incubated with 1:125 diluted Alexa Fluor488-conjugated donkey anti-mouse IgG (Jackson Immunoresearch Laboratories Inc., Westgrove, PA, USA) for 2h. After 4×10 min washes in TBST, the sections were mounted on gelatin-coated slides and coverslipped with Prolong-Gold antifade reagent (Cell Signaling Technology, Danvers, MA, USA).

For Gi-DREADD functional validation studies, PFC sections from dSAT-performing rats injected with either vehicle or CNO were processed for c-fos/CAMKII double immunohistochemistry. Briefly, sections were thawed and rinsed in 0.05 M Tris-buffered saline (TBS), followed by an incubation in TBS containing 0.03% H_2_O_2_ to block endogenous peroxidase. Following 1h incubation in 10% donkey serum, sections were incubated in rabbit anti-cfos antibody (SantaCruz Biotechnology, 1:2,000 dilution) for 48 h. Sections were then washed in TBST and incubated with biotinylated goat anti-rabbit (EMD Millipore Inc, Darmstadt, DE) for 2 hrs. Staining was developed by incubating sections in streptavidin-HRP followed by 3–3’-diaminobenzidine (DAB) and nickel chloride. After another incubation in 10% donkey serum, sections were incubated in anti-CamKII antibody (ThermoFisher, 1:1,000 dilution) for 48 h. Slices were then washed in TBST, incubated with biotinylated goat anti-mouse for 2h and developed by incubation in streptavidin-HRP followed by 3–3’-diaminobenzidine (DAB). Stained sections were mounted on gelatin-coated slides, air dried, dehydrated and coverslipped with DPX.

All sections were analyzed using a Leica brightfield/fluorescent microscope (DM4000B) equipped with DFC 425C digital camera and Leica Application Suite software (Leica Microsystems Inc.). The virus infection efficiency was evaluated by analyzing mCherry-CaMKII double-immunostaining in the anterior cingulate, prelimbic, and infralimbic regions from sections within 1mm of injection sites within the rostra-caudal space of mPFC. The virus infection efficiency was quantified by counting CaMKII-positive neurons with green fluorescence channel, and among those cells, the number of neurons co-expressing mCherry (red fluorescence channel) were counted. A cell was considered to be positive for a given marker if the corresponding signal was significantly above background fluorescence. In brief, images were captured using Image Overlay Module from both green and red filters simultaneously at 400x magnification from 3 representative fields in each hemisphere. The colocalization of mCherry with CamKII was confirmed with Confocal Laser Scanning microscope (Olympus Fluoview 3000RS). Approximately one hundred CaMKII-positive neurons (~50/hemisphere) from 2–3 fields per slice and a total of 3–5 sections/animal were examined. The percentage of cells coexpressing both the reporter (mCherry) and CaMKII from the total number of CaMKII-positive cells was then calculated for vector efficiency. A similar analysis was conducted to assess infection specificity by calculating the percentage of mCherry-positive cells that colocalize with CaMKII from the total number of cells expressing the reporter. For this analysis, approximately 100 mCherry-positive cells were examined per section in a given field.

For validation studies concerning functional efficacy of Gi-DREADD, images were captured in the brightfield microscopy mode and mPFC neurons from the performing and non-performing rats were analyzed for cfos-CaMKII double-immunostaining. The percentage of CaMKII-positive cells that colocalize with c-fos (a marker of neuronal activity) was calculated from the total number of CaMKII-positive cells following the vehicle and CNO injections. The cell counting parameters were similar to what were used for infection specificity and efficiency (see above). The effects of chemogenetic inhibition on performance associated changes in the activity of prefrontal output neurons was calculated by calculating the difference in CaMKII/c-fos colocalization in performing animals from colocalization group averages in non-performing animals in both manipulation conditions.

### *In vivo* amperometric recordings of cholinergic transmission

The effect of chemogenetic silencing of PFC projection neurons on top-down regulation of cholinergic signaling was assessed using *in vivo* amperometric recordings. For these experiments, a separate cohort of naïve young (N = 6) and aged (N = 5) rats underwent stereotaxic surgeries for bilateral infusions of Gi-DREADD vector as explained above. Approximately 4–5 weeks following vector infusions, animals were prepared for amperometric recordings of cholinergic transmission based on procedures described earlier (Howe et al., 2010; Parikh et al., 2014; Parikh et al., 2010; Yegla & Parikh, 2017). In brief, ceramic-based microelectrode arrays (Quanteon, Nicholasville, KY, USA) consisting of four rectangular platinum recording sites linearly arranged in pairs were coated with choline oxidase (Sigma-Aldrich, St. Louis, MO, USA). The enzyme was immobilized to the bottom pair of recording sites while the upper pair was coated with bovine serum albumin and served as sentinel channels. The electrode channels were electroplated with *m*-PD (m-phenylenediamine) to enhance the selectivity of choline against electroactive analytes such as ascorbic acid (AA), dopamine, and uric acid. Choline sensitivity and selectivity was tested for all electrodes by conducting an *in vitro* calibration. Microelectrode with a choline sensitivity >3pA/μM, limit of detection <400 nM, and selectivity ratio for choline:AA >50:1 were used for subsequent *in vivo* recordings.

Rats were anesthetized with urethane (1.0–1.25 g/kg, *i.p*.) and placed in a stereotaxic frame. Enzyme-coated microelectrodes were lowered into the mPFC (A/P: +3.0 mm, M/L: ±0.7 mm, D/V: −2.7–3.0 mm) of rats using a microdrive (MO-10, Narishige International, East Meadow, NY, USA). A reference electrode (Ag/AgCl) was implanted into the rostral cortical region of the contralateral hemisphere. Amperometric recordings were conducted at 2 Hz by applying a fixed potential of +0.7V, and data were digitized using a FAST-16 potentiostat (Quanteon). Background currents were stabilized for 60-min following which drug solutions were locally applied into the PFC using a glass capillary that was attached to the electrode and pulled to an internal tip diameter of 15–20 μm. For prefrontal chemogenetic manipulation, either vehicle (5% DMSO in 0.9% saline; 200 nL) or CNO (5 μM; 200 nL) was locally applied into the mPFC at 2–10 psi through the capillaries via a PTFE tubing connected to a picospritzer (ALA Scientific Instruments, Farmingdale, NY, USA). Following a 45-min drug exposure period, brief pulses of potassium (KCl 70mM; 200 nL) were applied locally into the mPFC to assess the magnitude of depolarization-evoked ACh release. Because prefrontal glutamatergic afferents project to the nBM/SI region (Zaborszky et al., 1997), and BF NMDA receptor activation is associated with tonic firing of cholinergic neurons (Khateb et al., 1995), we also assessed the impact of Gi-DREADD-induced suppression of prefrontal projection neurons on NMDA-induced tonic ACh release. For these experiments, an infusion cannula (30ga, Braintree Scientific, Braintree, MA, USA) filled with NMDA solution (40 μM NMDA, Sigma Aldrich) was implanted into the nBM/SI region of the BF (AP: −1.3 mm, ML: ±2.5 mm, and DV: −7.0 mm). Bicuculline (20 μM), a GABA A receptor antagonist, was also added into the solution to limit the influence of BF GABA interneurons on cholinergic activation. NMDA was infused into the BF at a rate of 1μL/min 45-min following the local application of either vehicle or CNO, and extracellular changes in choline levels were measured for 40 min. Choline signals were analyzed with respect to peak signal amplitudes. Self-referencing was adopted to eliminate any artifacts on enzyme-coated channels due to background noise levels or drug application by subtracting currents from sentinel channels. Amperometric recording data for KCl-elicited signals were binned at 0.5 s. BF NMDA-evoked signals were box-car filtered by a moving average of 20 data points and binned at 1-min. All data were expressed as the average of three signals per manipulation per animal. Prefrontal recordings occurred in both hemispheres and counterbalancing for local application of drugs was employed across hemispheres and age groups.

### Statistical analysis

Statistical analyses were performed using SPSS/PC+ version 26.0 (IBM-SPSS, Armonk, NY, USA). Pre-surgery behavioral data for hits, correct rejections, SAT scores and total omissions were analyzed using one-way ANOVAs to compare group differences. Post-surgery behavioral data for dSAT scores, hits and correct rejections, were analyzed using mixed factor repeated measures ANOVA with block (3 levels) or signal duration (3 levels), and vehicle/CNO manipulation (2 levels) as within-subject variables, and age (2 levels) as between-subject variables. When appropriate, one-way ANOVAs and Fisher’s least significant difference (LSD) tests were used for *post hoc* comparisons to determine the source of interactions. Post-doc comparisons for within-subject variables were conducted using paired *t*-tests. The effects of age and Gi-DREADD manipulations on cortical ACh release were analyzed using 2 × 2 ANOVAs. All immunohistochemistry data were analyzed using one-way ANOVA. A cut-off *p* value ≤0.05 was considered statistically significant.

## Results

### Vector efficacy and functional validation

**Figure 2.**
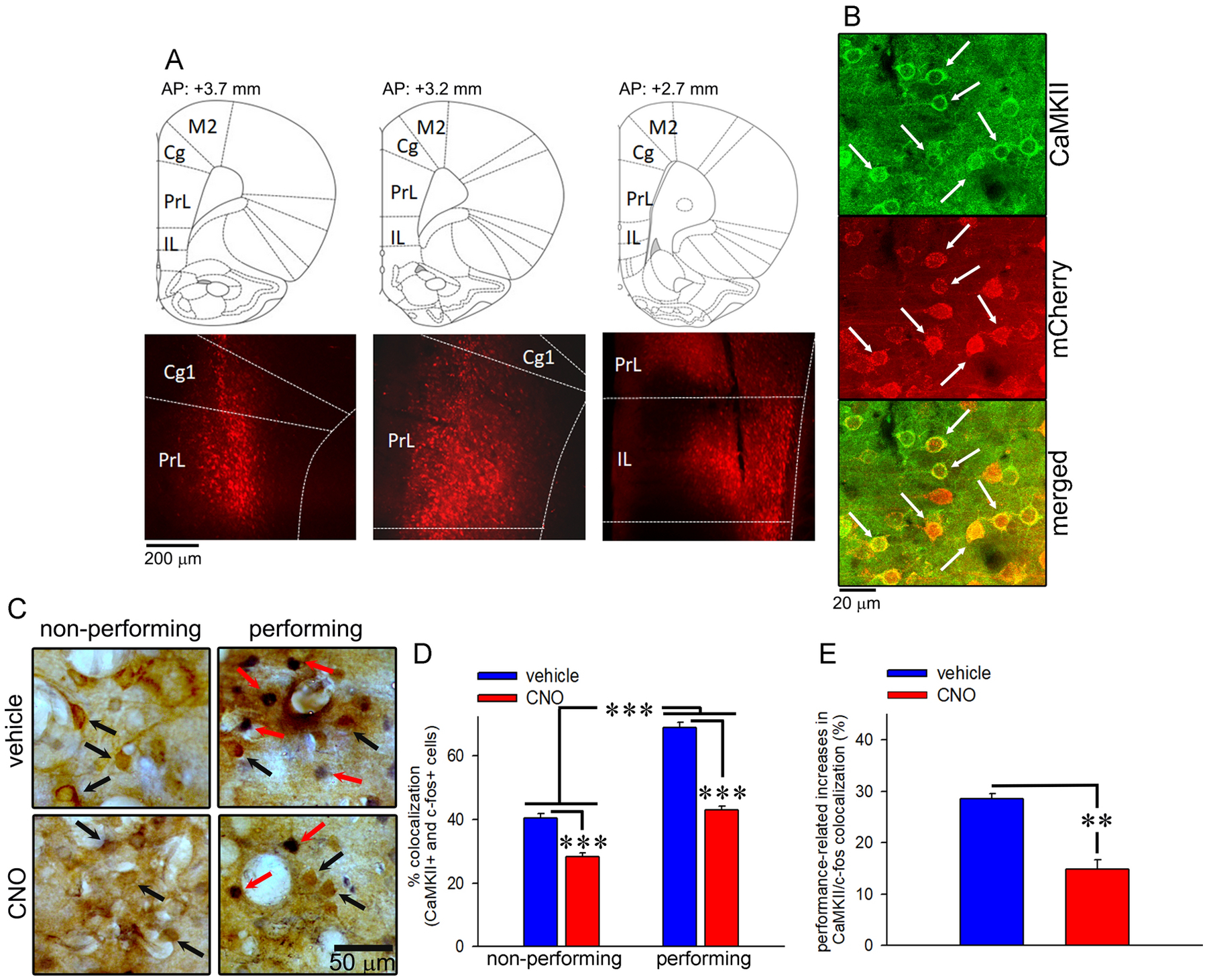
Vector efficacy and functional validation. A) mCherry-expressing neurons depict the spread of AAV vector along the rostral-caudal axis of medial prefrontal cortex (mPFC). Serial sections show vector expression in the cingulate (cg), prelimbic (PrL), and infralimbic (IL) subregions of the mPFC. B) Representative confocal images from the sampled area depicting CaMKII-expressing cells (green), mCherry-expressing cells (red), and their colocalization (yellow; merged images) confirm the targeting of prefrontal projection neurons by the Gi-DREADD vector. The colocalization of CaMKII with mCherry is marked with white arrows. C) Representative images depicting CaMKII/c-fos double immunostaining in non-performing and performing rats. CaMKII+ cells are marked with black arrows while their colocalization with c-fos+ cells is marked with red arrows. D) The percentage of CaMKII/c-fos colocalization was markedly higher in performing rats. Moreover, CNO administration reduced colocalization in both groups; however, the effects were more dramatic in performing animals. E) Performance-associated increases in c-fos activity detected in CaMKII-positive neurons significantly declined following CNO injection, confirming the functional efficacy of the chemogenetic strategy used to silence prefrontal projection neurons. **, *** *p* < 0.01, 0.001.

The extent of AAV vector spread, as demonstrated by mCherry expression, along the rostral-caudal axis of mPFC that include regions of cingulate, prelimbic and infralimbic cortex is illustrated in Fig 2A. Quantitative analyses of CaMKII/mCherry colocalization that reflect the infection efficiency of AAV vector expressing Gi - DREADD in prefrontal projection neurons revealed that the expression efficiency ranged from 69 – 73 % (Fig 2B). The degrees of colocalization did not differ between young (71.10 ± 0.87%) and aged (69.84 ± 5.52%) animals (*F* (1, 15) = 1.76, *p* = 0.204). The mCherry-positive neurons were also separately evaluated for infection specificity. Based on this assessment, CaMKII was colocalized with mCherry in 90.02 ± 2.64% of infected cells. Given the vector construct was designed to express Gi-DREADD tagged with mCherry under the control of CaMKII promoter, these data illustrate that infection was promoter-selective.

The functional efficacy of Gi-DREADD-induced suppression of activity of prefrontal projection neurons was evaluated by comparing CaMKII/cFos double immunostaining in non-performing and SAT-performing young rats exposed to either a systemic injection of vehicle or CNO prior to the dSAT testing session. It should be noted that performing rats used for these validation studies attained SAT criterion as described in the behavioral methods for the DREADD behavior studies. In general, performing animals, compared to their non-performing counterparts, demonstrated increased CaMKII/cFos colocalization following both injection types, indicating that the activity of mPFC projection neurons was dependent on attention task performance (main effect of performance: *F* (1, 8) = 338.89, *p* < 0.001; Fig 2C and 2D). In addition, CNO manipulation reduced colocalization in both groups as compared to the vehicle condition; although the effects were more dramatic in performing animals (main effect of manipulation: *F* (1, 8) = 258.98, *p* < 0.001; Fig 2C and 2D). The performance-associated increases in c-fos activity detected in CaMKII-positive neurons declined significantly following the CNO injection as compared to the vehicle injection (CNO: 14.81 ± 1.84%, vehicle: 28.50 ± 1.04%; *F* (1, 4) = 23.70, *p* < 0.01; Fig 2E). Collectively, these results confirm functional efficacy of the chemogenetic strategy used to silence prefrontal projection neurons recruited during the attention task in our study.

### Pre-surgery SAT performance

**Figure 3.**
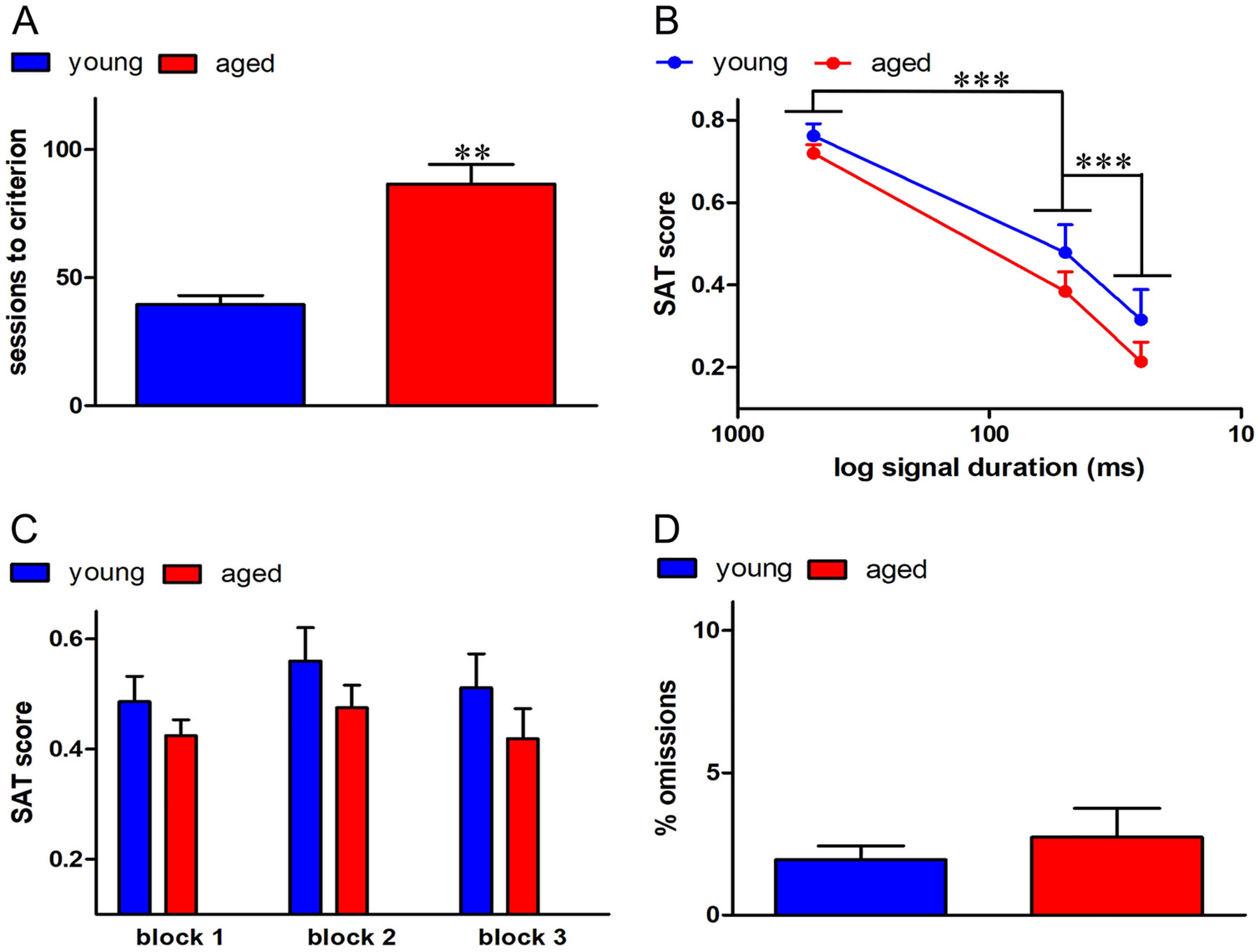
Pre-Surgery task performance. A) Aged rats required significantly more training sessions to reach criterion in SAT as compared to the young animals. B) Attention performance was dependent on signal duration as expected. C) Average SAT scores did not differ by age group, with no significant differences across blocks and no block × age interactions. D) Omissions remained low and did not significantly differ between age groups. Together, these data confirm that attentional performance prior to vector infusion and vehicle/CNO injection remained similar in all groups *** *p* < 0.001 (pairwise comparisons for main effect of signal duration).

Aged rats required significantly more training sessions to reach criterion in SAT as compared to the young animals (aged: 86.67 ± 7.56; young: 39.50 ± 3.93; *F* (1, 15) = 28. 34, *p* < 0.01; Fig 3A). However, SAT performance after the attainment of criterion and prior to surgeries, remained comparable between young and aged rats. As expected, attention performance remained signal duration-dependent (*F* (2, 30) = 73.52, *p* < 0.001; pairwise comparisons of SAT scores for main effect of signal: *p* < 0.001 for both 500ms vs 50 ms and 50 ms vs 25 ms; Fig 3B), but this measure neither differed as a function of age nor interacted with signal duration (*p* > 0.43 for both main effect of age and age × signal duration interactions). Average SAT scores remained similar across the blocks (*F* (2, 30) = 0.51, *p* = 0.60; Fig 3C) and block × age interactions remained insignificant (*F* (1,15) = 0.12, *p* = 0.60). Performance on both signal and non-signal trials did not differ between the two groups (% hits: *F*(1, 15) = 0.61, *p* = 0.45; % correct rejections: *F* (1, 15) = 0.20, *p* = 0.66). Additionally, omissions remained low and similar in both groups (*F*(1, 15) = 0.84, *p* = 0.37; Fig 3D). Similarly, SAT performance after surgery and prior to dSAT testing remained comparable between young and aged rats (*F* (1, 15) = 0.12, *p* = 0.73). These data confirm that attentional performance prior to Gi-DREADD vector infusion as well as vehicle/CNO injection remained similar in all groups.

### dSAT performance

**Figure 4.**
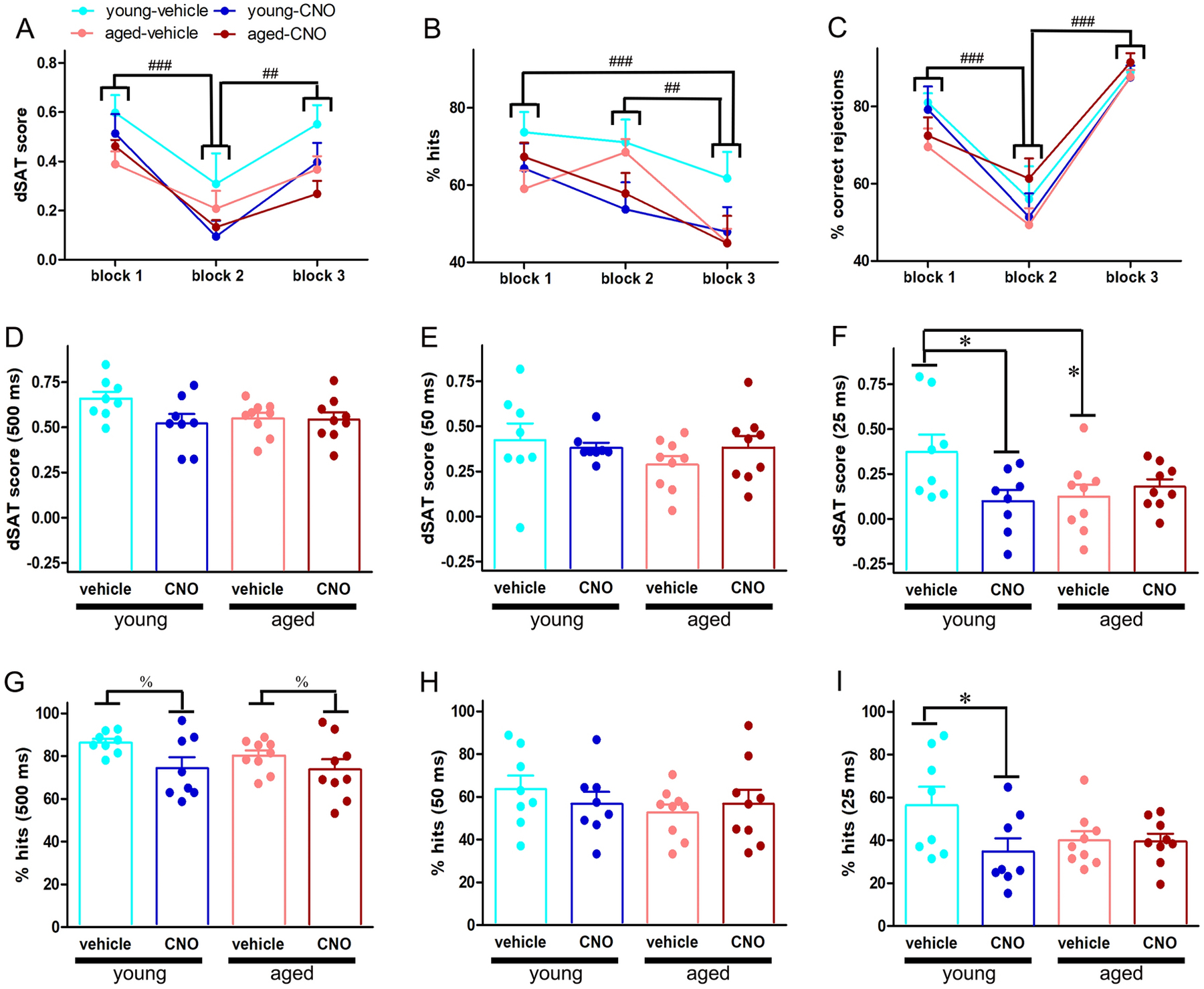
Effects of chemogenetic silencing of PFC output neurons on the dSAT performance. A) As expected, performance declined significantly in the distractor block as compared to the pre- and post-distractor blocks in both age groups. B) The distractor effects on attentional performance included detriments in response accuracies on signal trials (i.e. hits). C) In addition, attentional performance on non-signal trials (i.e. correct rejections) was also impaired. D & E) Overall dSAT scores did not differ across groups or injection type on 500ms or 50ms trials. F). Young animals displayed a drastic decrease in dSAT scores for 25ms in response to CNO, but such decrements in performance were not observed in aged animals. G) Response accuracies on signal trials (i.e. hits) were significantly decreased in response to CNO in both young and aged animals on 500ms trials. H) Percentage of hits did not differ across groups or injection type on 50ms signal trials. I) Similar to overall dSAT scores, response accuracies on 25ms signals reduced significantly in young rats but not aged rats following CNO injection. ##, ### *p* < 0.01, 001 (pairwise comparisons for main effect of block; % (main effect of manipulation), *, ** *p* < 0.05, 0.01 (post hoc tests).

Because visual distractors impose higher attentional load and recruit PFC-mediated top-down mechanisms to maintain performance (Paneri & Gregoriou, 2017; Sarter et al., 2006), we investigated how attentional performance under distracting conditions might be impacted by Gi-DREADD-induced silencing of PFC projection neurons in young and aged animals. The dSAT scores differed significantly across blocks (main effect: *F* (2, 30) = 22.84, *p* < 0.001) with performance declining robustly in the distractor (2^nd^) block as compared to the pre- and post-distractor blocks (pairwise comparisons for block effect: *p* < 0.001 block 2 vs. block 1; *p* = 0.01 block 2 vs. block 3; Fig 4A). As expected, the distractor effects on overall attentional performance involved detriments in response accuracies on both signal trials (main effect of block on hits: *F* (2, 30) = 14.09, *p* < 0.001; Fig 4B) and non-signal trials (main effect of block on correct rejections: *F* (2, 30) = 50.86, *p* < 0.001; Fig 4C). Significant decline in the proportion of correct rejections but not hits in the distractor block indicated performance decrements during the presentation of visual distractors were primarily driven by higher false alarms (hits: *p* > 0.33, block 2 vs block 1; correct rejections: *p* < 0.001, block 1 vs block 2). On the other hand, performance recovery in the post-distractor block mostly occurred due to higher response accuracies in the non-signal trials, (i.e. correct rejections); hit rate remained substantially lower in this block (see pairwise comparisons for hits and correct rejections in Fig 4B and C). The effects of CNO manipulation on hits and correct rejections did not reach significance (both *p* > 0.09). Moreover, no significant block × age × CNO manipulation was observed for these measures (both *p* > 0.50).

Mixed factor ANOVA on dSAT performance showed a significant main effect of signal duration (*F* (2, 30) = 64.69, *p* < 0.001). Although the main effect of CNO manipulation was not significant (*F* (1, 15) = 1.83, *p* = 0.20), a significant 3-way interaction of these variables with age was observed (signal duration × manipulation × age: *F* (2, 30) = 3.26, *p* = 0.05). To find the source of this interaction, separate 2×2 ANOVA were conducted for each signal duration (Fig 4D–F). These analyses revealed a significant age × CNO manipulation interaction for dSAT scores at the 25ms signal (*F* (1, 15) = 8.59, *p* = 0.01). Post hoc comparisons show when prefrontal functioning remained intact, aged animals performed poorly as compared to their young counterparts (*p* = 0.04 young vehicle vs aged vehicle; Fig 4F). In response to CNO injection, young animals displayed a drastic decrease in dSAT scores for 25ms signal as compared to the vehicle injection (*p* = 0.01; Fig 4F). Surprisingly, such decrements in performance were not observed in aged animals (*p* = 0.48, aged vehicle vs aged CNO). The proportion of hits analyzed for each signal duration in the dSAT session exhibited similar trends (Fig 4G–I). Response accuracies on 25 ms signal reduced significantly in young rats but not aged rats following CNO injection (age × CNO manipulation interaction: *F* (1,15) = 12.57, *p* = 0.003; young: *p* = 0.01 CNO vs vehicle; aged: *p* = 0.94 CNO vs vehicle; Fig 4I). dSAT scores and hits at higher signal durations (500ms and 50 ms, respectively) remained variable and were neither significantly impacted by age nor did this effect interact with CNO manipulation (all *p* > 0.05; Fig 4D, E, G, H). Although % hits for 500ms signal were marginally but significantly reduced by CNO injection (main effect: *F* (1,15) = 5.69, *p* = 0.03; Fig 4G), the effects on dSAT scores at this signal did not reach significance (*p* = 0.16; Fig 4D) presumably due to higher variability in the non-signal trial performance. Together, these data illustrate the deleterious effect of prefrontal inhibition under distracting conditions was more prominent in young animals than aged subjects.

The dSAT performance of young performing rats used in DREADD functional validation studies remained comparable to performance of young rats used in DREADD behavior studies under vehicle condition (dSAT scores: 0.41 ± 0.02 vs. 0.48 ± 0.07; *F* (1,9) = 0.39, *p* = 0.54). Likewise, dSAT performance under the CNO condition remained insignificant between the two cohorts of animals (*F* (1,9) = 1.36, *p* = 0.27). Together, these data illustrate that dSAT performance of animals used in DREADD validation studies was representative of the overall performance of young rats that underwent chemogenetic manipulations.

To determine whether the behavioral effects of CNO observed in young rats were specific to Gi-DREADD-mediated silencing of mPFC output neurons, dSAT performance of DREADD-naïve young performing rats was examined. These animals were infused with the control AAV vector expressing mCherry under the control of CaMKIIa promoter. A repeated measures ANOVA revealed neither a significant effect of CNO manipulation (*F* (1,12) = 0.81, *p* = 0.79), nor there was a manipulation × signal duration (*F* (2,12) = 0.20, *p* = 0.82) or manipulation × block (*F* (2,12) = 2.71, *p* = 0.11) interaction in these animals. The comparison of dSAT performance by vector type revealed CNO manipulation resulted in significantly lower dSAT scores and lower proportion of hits (both *p* < 0.05); however, these behavioral measures remained comparable between the two vectors following vehicle manipulation (Supplementary Fig 1). Together, these data indicate that CNO-mediated effects on behavior were DREADD-specific.

### Chemogenetic silencing of prefrontal projection neurons and prefrontal cholinergic signaling

**Figure 5.**
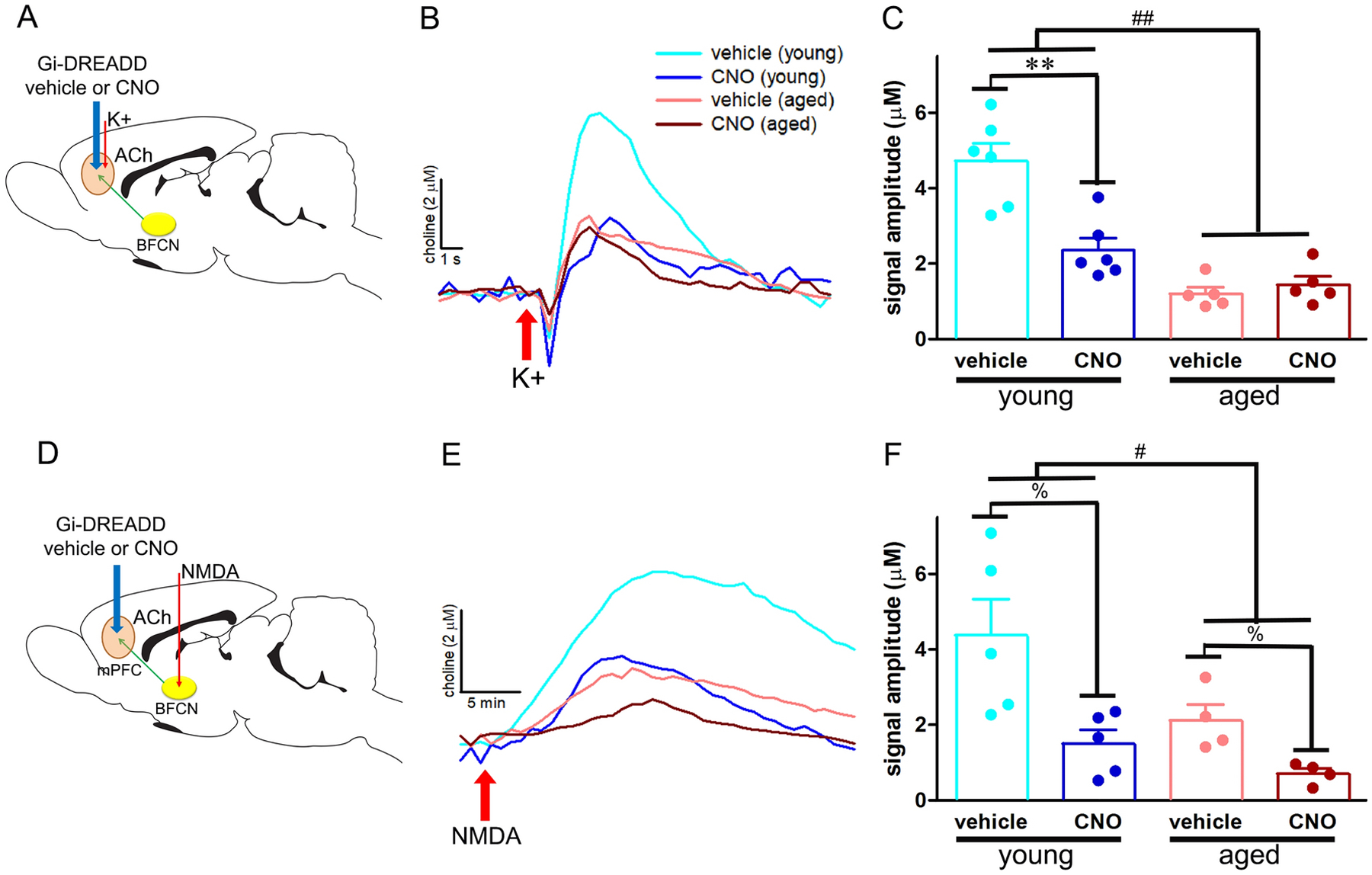
Effects of **c**hemogenetic silencing of prefrontal projection neurons on prefrontal cholinergic signaling. A) Schematic illustrating the experimental manipulation to assess phasic ACh release from mPFC-projecting BF cholinergic neurons (BFCN) following local injection of either vehicle or CNO. B) Representative traces depicting K^+^-evoked cholinergic transients from young and aged rats with intact or silenced PFC –projection neurons. C) The amplitudes of cholinergic transients were significantly reduced in aged animals and in response to CNO manipulation in young animals; depolarization-evoked cholinergic signals in aged animals were comparable regardless of injection. D) Schematic illustrating the experimental manipulation to assess tonic ACh release from mPFC-projecting BFCN following local injection of either vehicle or CNO. E) Representative boxcar filtered traces depicting minute-based changes in extracellular choline concentration from young and aged rats with intact or silenced PFC projection neurons following BF NMDA infusions. F) NMDA-evoked cholinergic signal amplitudes significantly reduced with both CNO injection and with age. ##, ### *p* < 0.01, 001 (pairwise comparisons for main effect of block; % (main effect of manipulation), *, ** *p* < 0.05, 0.01 (post hoc tests)

The results on amperometric recordings of cholinergic transmission are summarized in Fig 5A–F. The amplitudes of depolarization-evoked cholinergic transients were significantly reduced by age (main effect: *F* (1, 9) = 26.84, *p* < .01), CNO manipulation (main effect: *F* (1, 9) = 26.15, *p* < 0.010), and there was a significant interaction between the two factors (age × manipulation interaction: *F* (1, 9) = 38.73, *p* < 0.001). Post hoc comparisons exemplified that young animals were particularly susceptible to Gi-DREADD-mediated silencing of PFC projection neurons as indicated by robust reduction in the amplitudes of KCl-elicited cholinergic signals following CNO application (*p* = 0.001 vs vehicle; Fig 5B,C). On the other hand, depolarization-evoked ACh release in aged animals was comparable regardless of injection (p = 0.21 vehicle vs CNO) further suggesting that the main effect of CNO manipulation on reduction in cholinergic transients was primarily driven by young animals.

BF NMDA infusions produced min-based tonic increases in cholinergic transmission in the mPFC (Fig. 5E). NMDA-evoked cholinergic signal amplitudes significantly reduced with both CNO injection and with age (main effect of manipulation: *F* (1,7) = 8.17, *p* = 0.02; main effect of age: *F* (1,7) = 12.25, *p* = 0.01; Fig 5F). However, the effects of age on signal amplitudes did not interact with CNO manipulation (age × manipulation interaction: *F* (1,7) = 0.96, *p* = 0.36). These data illustrate that chemogenetic inhibition of PFC projection neurons produced disruption in top-down control of tonic cholinergic signaling in young and aged rats to a similar extent.

## Discussion

Although deficits in both the structure and function of PFC are commonly observed in aging, paradoxical age-related increases in PFC activity that correlate with improved performance in a variety of cognitive tasks have also been reported in functional neuroimaging studies (Cabeza et al., 2004; Davis et al., 2008; Grady et al., 1994). On the other hand, several human studies have not found significant performance improvements with elevated PFC activity in aging (Rypma et al., 2007; Stevens et al., 2008; Zarahn et al., 2007). Therefore, it remains debated whether age-related changes in PFC activity and associated recruitment of neural processes reflect beneficial, detrimental, or non-specific neural adaptations that do not have functional consequences. Moreover, the impact of PFC reorganization on cholinergic transmission, which is important for top-down allocation of attentional resources and optimization of signal detection (Sarter et al., 2005), in aging remains unknown. The present experiments sought to determine if attentional capacities and cholinergic signaling in aging were more dependent on PFC output, an area hypothesized to increase activity in aging to compensate for inefficacies in bottom-up neural processing networks.

As noted earlier, the dSAT operant task employed in our study requires the engagement of top-down cognitive control mechanisms to maintain attentional performance (Demeter et al., 2008; Nuechterlein et al., 2009). These mechanisms include filtering irrelevant information presented in the form of visual distractors, facilitating processes that discriminate signals from non-signals under challenging conditions, and sustaining performance by detecting unpredictably occurring signals of varying signal durations. Silencing of PFC output neurons-expressing Gi-DREADDs with CNO in young rats robustly disrupted the ability to discern signals of lowest intensity from non-signal events. These results are consistent with the evidence that the PFC is critically involved in top-down modulation of neural processes required for delegating attentional resources to selective stimuli and ignoring irrelevant distractions (Chao & Knight, 1995; Gazzaley et al., 2007; Lee & D’Esposito, 2012; Mesulam, 1981; Paneri & Gregoriou, 2017; Zanto et al., 2011). Moreover, dSAT scores for 25ms signal remained substantially lower in aged rats as compared to the young counterparts under control (vehicle injection) conditions, illustrating deficits in age-related top-down control of attention as suggested earlier (Madden, 2007; Quigley & Müller, 2014).

Surprisingly, the dSAT performance in aged rats remained insensitive to the chemogenetic silencing PFC projection neurons. It has recently been argued that increased PFC recruitment in aging does not support compensation, but rather is reflective of reduced efficiency (Morcom & Henson, 2018). Moreover, altered prefrontal activity in aging is proposed to represent a discordance between available cognitive resources and task demands, which may not necessarily indicate successful compensation (i.e. improved performance), but could also reflect either unsuccessful compensation (i.e. impaired performance) or non-specific recruitment with no functional consequences (Cabeza & Dennis, 2012). Therefore, our findings are suggestive of inefficient PFC output in aging that is unable to compensate, and rather, loses its ability to modulate cortical and subcortical regions to optimize attention under challenging conditions. In other words, aged rats might rely less on PFC selectivity to regulate attentional control, and rather, recruit non-specific cortical regions that alter behavioral strategies in the face of declining attentional capacities. This interpretation is consistent with the dedifferentiation hypothesis of aging (Dennis & Cabeza, 2011; Koen & Rugg, 2019). Because diminished dSAT performance in aged rats did not further worsen with PFC silencing, it is possible that reorganization of other cortical regions in the attentional network may compensate for reduced PFC output in aging.

The BF cortical cholinergic input system is a critical component of the brain’s attentional system (Everitt & Robbins, 1997; Sarter et al., 2005; Sarter & Parikh, 2005). Specifically, cholinergic inputs to the PFC are recruited with increased attentional load, and deafferentation of these inputs disrupts top-down control of attention (Dalley, Theobald, et al., 2004; Newman & McGaughy, 2008). Recent evidence suggests that phasic cholinergic transients in the PFC mediate the detection of attention-demanding cues by switching perceptual processing of the stimulus to stimulus-evoked activation of response rules (Gritton et al., 2016; Howe et al., 2013; Parikh et al., 2007). In contrast, tonic increases in cortical ACh release is hypothesized to reflect top-down cholinergic neuromodulation that facilitate signal detection under conditions of high attentional load and distractor challenges (Hasselmo & Sarter, 2011; St Peters et al., 2011). Infusions of NMDA into the BF produced slow (i.e. minute-based) increases in extracellular ACh levels in the PFC in our study, which is in agreement with previous investigations that demonstrate tonic firing of BF cholinergic neurons and long-lasting changes in cortical ACh efflux (Fadel et al., 2001; Khateb et al., 1995). Consistent with previous investigations, an age-dependent decrease in both the NMDA-mediated tonic and depolarization-evoked phasic ACh release was apparent, providing further support for the causal role of cholinergic signaling in mediating attentional performance in aging (Fadel, 2011; Parikh et al., 2014; Parikh et al., 2013).

Interestingly, while suppression of PFC projection neurons resulted in attenuation of tonic cholinergic signaling across age groups, the magnitude of phasic cholinergic transients was reduced only amongst young animals. These results parallel our behavioral findings that response accuracies to 500ms signals were significantly reduced in both young and aged rats following CNO-injections in the dSAT test session; however, such decrements in response accuracies and overall measure of attention were not observed at lower signal durations in aged rats. Although the relationship between the two modes of cholinergic signaling is not entirely clear, tonic cholinergic modulation is postulated to increase the probability and magnitude of cholinergic transients to facilitate signal detection under distracting conditions (Hasselmo & Sarter, 2011; Parikh & Sarter, 2008). Given the direct anatomic links and long-loop transynaptic interactions between PFC glutamatergic output neurons and BF neurons in the nBM/SI region (Gielow & Zaborszky, 2017; Sarter et al., 2005), it can be speculated that dSAT performance deficits in CNO-injected young rats might plausibly be linked to disrupted PFC-mediated tonic cholinergic modulation of fast cholinergic transients. While diminished PFC-mediated top-down control of cholinergic signaling may explain age-related deficits in attentional capacities, the reduction in the magnitude of cholinergic transients in naïve aged rats remained comparable between the vehicle and CNO condition. As noted earlier, the dSAT performance did not further exacerbate in aged rats following PFC silencing. These results raise the intriguing possibility that perhaps the two cholinergic signaling modes and their interactions are differentially regulated when PFC function is compromised in aging. Electrophysiological studies have indeed demonstrated the presence of two separate populations of cholinergic neurons in the BF that showing distinct firing (i.e. phasic and tonic) patterns and function-specific recruitment (Laszlovszky et al., 2020; Unal et al., 2012). Thus, it is plausible that loss of PFC efficiency and consequent activation of non-specific or complimentary cortical regions in aging may differentially recruit BF cholinergic neurons to impact attentional networks. This view aligns with our recent observation that cholinergic signaling modulate the dynamics of reorganized cortical circuits to preserve attentional capacities in aging (Yegla et al., 2021). However, further studies are warranted to discern the validity of such hypotheses.

While our research provides important insights into age-related shifts in prefrontal control of attention, some limitations pertaining to the study design and approach require discussion. First, the use of CNO as a DREADD agonist may exert off-target effects that could confound our results, and we acknowledge this could be a limitation. However, a recent study did not find any effects of systemic CNO administration on attentional performance in 5-choice serial reaction time task in control mice at doses higher than what was used in our study (Jendryka et al., 2019). Moreover, intracranial CNO injections in several brain regions including the cortex, hippocampus, and ventral tegmental area did not produce DREADD-independent behavioral effects (Ge et al., 2017; Lichtenberg et al., 2017; Mahler et al., 2014). Therefore, it is likely that the behavioral and neurochemical effects observed due to CNO administration are DREADD-specific, at least at the doses used in our study. Our data from the control vector experiments, which did not reveal any significant effects of CNO on dSAT performance in DREADD-naïve young performing rats, further support this conclusion. Second, the current experiments employed a between-subjects design, such that separate cohorts of young and aged animals were used for behavioral and electrochemical analysis on cholinergic transmission. Therefore, the interpretations concerning the involvement of PFC-mediated top-down regulation of cholinergic mechanisms in the observed age-related differences in cognitive effects remain speculative. Specifically, it is possible that the effects of chemogenetic suppression of PFC projection neurons on age-related differences in cholinergic signaling may not fully translate to alterations in top-down attentional capacities. To address this limitation, further studies should be designed to determine the impact of chemogenetic manipulation targeting prefrontal projections to the BF in young and aged rats on the cholinergic-attention system. Third, CNO was administered through different routes across the behavior and amperometric recording experiments (i.e. systemic vs local, respectively). Here, it should be noted that previous studies on DREADD-specific manipulations of LC noradrenergic neurons and ventral pallidal neurons show comparable differences in the firing frequency and behavioral output following systemic and intracranial injections of CNO (Mahler et al., 2014; Vazey & Aston-Jones, 2014). Moreover, the effects of CNO are suggested to be DREADD-specific regardless of the route of CNO administration (Mahler & Aston-Jones, 2018; Smith et al., 2016). However, we cannot rule out differences in the concentration of CNO at local receptors between the two routes, which may potentially affect Gi-DREADD-specific suppression of mPFC output neurons. Fourth, the aged rats used in our study were retired breeders, while young animals were sexually naïve, which could be a potential confounding factor in the study’s design. Indeed, long term exposure to sexual experience can reportedly stimulate hippocampal neurogenesis and improve novel object recognition performance in middle aged rats as compared to virgin age-matched control rats (Glasper & Gould, 2013). Additionally, performance differences in certain behavioral paradigms has been observed between retired breeder and virgin aged mice (Ingram et al., 1983). Although it remains unclear if sexual experience can impact age-related changes in PFC recruitment and top-down cognitive control, we cannot rule out the possibility that some of the age-related differences in attention performance and PFC cholinergic activity following our DREADD manipulation might be related to prior sexual experience. Lastly, this study was conducted only in male rats, which makes it difficult to generalize functional consequences of age-related PFC reorganization to both sexes.

In summary, our results for the first time show differential impact in young and aged rats of chemogenetic PFC silencing on attention and cholinergic transmission. Our findings suggest the reduced efficiency of the PFC to recruit top-down mechanisms may be associated with attentional impairments under challenging conditions and disrupted activation of the BF cholinergic projection system. Additionally, alterations in the activity of PFC output neurons may not necessarily reflect compensation in aging. Furthermore, reducing PFC output in aging does not worsen attentional performance, potentially reflecting activation of complimentary cortical networks to maintain performance. As noted earlier, attentional capacities often decline with advancing age; however, the rate and degree of such decline can vary substantially between individuals (Fortenbaugh et al., 2015; Verhaeghen & Cerella, 2002). A subset of elderly individuals remain resilient to such impairments, particularly those who display certain epidemiological factors (i.e. healthy diet, increased physical activity, elevated education) (Gu et al., 2018; Roldan-Tapia et al., 2012; Stern, 2012). Conversely, some individuals display profound attentional deficits that progress at significantly elevated rates in comparison to age-matched peers (McDonough et al., 2019; McMurtray et al., 2006). Given the high vulnerability of the cholinergic-attention system and PFC function in aging (Burk et al., 2002; Parikh et al., 2013; Samson & Barnes, 2013), it is possible that differences in neuroadaptive capacity (i.e. to successfully compensate for such age-related functional decrements) may underlie individual differences in attentional control in older adults.

## Supplementary Material

1727859_Supp_info
