# Supplementary material for: Chemogenetic inhibition of prefrontal projection neurons constrain top-down control of attention in young but not aged rats
Source: Brain Struct Funct. Author manuscript; Available in PMC 2022 Sep 1. (PMC8355172; doi:10.1007/s00429-021-02336-2)
Supplement: 1727859_Supp_info [file NIHMS1727859-supplement-1727859_Supp_info.docx]

**Supplementary Information**

**Supplementary Figure 1**

**
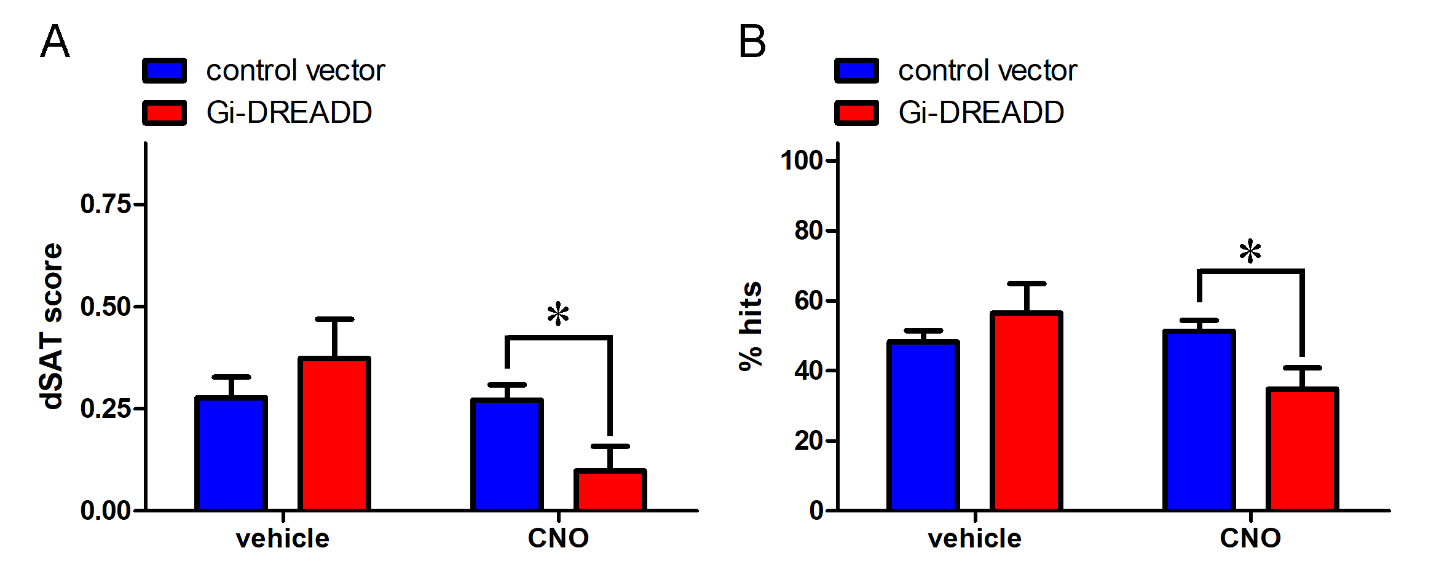
**

**Supplementary figure 1.** DREADD-specific performance effects in young rats. Animals were trained in the sustained attention task (SAT) to criterion and received injections of the control vector (AAV vector expressing mCherry reporter under the control of CaMKIIa promoter) and Gi-DREADD vector (AAV vector expressing both hM4D(Gi) and mCherry under the control of CaMKIIa promoter) into the PFC, respectively. All animals received systemic injections of vehicle and CNO as a manipulation using a counter-balanced design prior to testing in the distractor (dSAT) session. A 2x2 ANOVA revealed a main effect of manipulation and a vector x manipulation interaction for both the dSAT scores (main effect: *F*(1,13) = 7.70, *p* = 0.016; interaction: *F*(1,13) = 7.24, *p* = 0.019; Fig 1A) and % hits (main effect: *F*(1,13) = 5.18, *p* = 0.040; interaction: *F*(1,13) = 9.44, *p* = 0.009; Fig 1B) at the shortest (i.e. 25 ms) signal duration. Post hoc tests showed the performance remained comparable following vehicle manipulation between the two vectors (both measures: *p* ≥ 0.41). However, CNO injection significantly impaired performance in animals infused with the Gi-DREADD vector as compared to the control vector (dSAT score: *p* = 0.039; % hits: *p* = 0.036; Fig 1A and 1B). All data are represented as means ± SEM. * *p* < 0.05
